# Supplementary material for: The Effects of Radioligand Therapy on Quality of Life and Sexual Function in Patients with Neuroendocrine Neoplasms
Source: Cancers (Basel). 2022 Dec 24;15(1):115. doi: 10.3390/cancers15010115 (PMC9817532; doi:10.3390/cancers15010115)
Supplement: Supplementary file 1 [file cancers-15-00115-s001.zip › cancers-2090559-supplementary.pdf]

1. **MeSH terms and keywords used for literature search:**

((("quality of life"[MeSH Terms] OR ("quality"[All Fields] AND "life"[All Fields]) OR "quality of life"[All Fields]) AND ("neuroendocrin"[All Fields] OR "neuroendocrinal"[All Fields] OR "neuroendocrines"[All Fields] OR "neurosecretory systems"[MeSH Terms] OR ("neurosecretory"[All Fields] AND "systems"[All Fields]) OR "neurosecretory systems"[All Fields] OR "neuroendocrine"[All Fields])) OR (("receptors, peptide"[MeSH Terms] OR ("receptors"[All Fields] AND "peptide"[All Fields]) OR "peptide receptors"[All Fields] OR ("peptide"[All Fields] AND "receptor"[All Fields]) OR "peptide receptor"[All Fields]) AND ("radioisotopes"[MeSH Terms] OR "radioisotopes"[All Fields] OR "radionuclide"[All Fields] OR "radionuclides"[All Fields] OR "radionuclid"[All Fields] OR "radionuclide s"[All Fields] OR "radionuclidic"[All Fields] OR "radionuclidically"[All Fields] OR "radionuclids"[All Fields]) AND ("therapeutics"[MeSH Terms] OR "therapeutics"[All Fields] OR "therapies"[All Fields] OR "therapy"[MeSH Subheading] OR "therapy"[All Fields] OR "therapy s"[All Fields] OR "therapys"[All Fields]) AND ("quality of life"[MeSH Terms] OR ("quality"[All Fields] AND "life"[All Fields]) OR "quality of life"[All Fields])) OR ("PRRT"[All Fields] AND ("quality of life"[MeSH Terms] OR ("quality"[All Fields] AND "life"[All Fields]) OR "quality of life"[All Fields])) OR (("radioligand"[All Fields] OR "radioligand s"[All Fields] OR "radioligands"[All Fields]) AND ("therapeutics"[MeSH Terms] OR "therapeutics"[All Fields] OR "therapies"[All Fields] OR "therapy"[MeSH Subheading] OR "therapy"[All Fields] OR "therapy s"[All Fields] OR "therapys"[All Fields]) AND ("quality of life"[MeSH Terms] OR ("quality"[All Fields] AND "life"[All Fields]) OR "quality of life"[All Fields])) OR ("RLT"[All Fields] AND ("quality of life"[MeSH Terms] OR ("quality"[All Fields] AND "life"[All Fields]) OR "quality of life"[All Fields])) OR (("sexual behavior"[MeSH Terms] OR ("sexual"[All Fields] AND "behavior"[All Fields]) OR "sexual behavior"[All Fields] OR "sexual"[All Fields] OR "sexually"[All Fields] OR "sexualities"[All Fields] OR "sexuality"[MeSH Terms] OR "sexuality"[All Fields] OR "sexualization"[All Fields] OR "sexualize"[All Fields] OR "sexualized"[All Fields] OR "sexualizing"[All Fields] OR "sexuals"[All Fields]) AND ("functional"[All Fields] OR "functional s"[All Fields] OR "functionalities"[All Fields] OR "functionality"[All Fields] OR "functionalization"[All Fields] OR "functionalizations"[All Fields]

OR "functionalize"[All Fields] OR "functionalized"[All Fields] OR "functionalizes"[All Fields] OR "functionalizing"[All Fields] OR "functionally"[All Fields] OR "functionals"[All Fields] OR "functioned"[All Fields] OR "functioning"[All Fields] OR "functionings"[All Fields] OR "functions"[All Fields] OR "physiology"[MeSH Subheading] OR "physiology"[All Fields] OR "function"[All Fields] OR "physiology"[MeSH Terms]) AND ("neuroendocrine tumour"[All Fields] OR "neuroendocrine tumors"[MeSH Terms] OR ("neuroendocrine"[All Fields] AND "tumors"[All Fields]) OR "neuroendocrine tumors"[All Fields] OR ("neuroendocrine"[All Fields] AND "tumor"[All Fields]) OR "neuroendocrine tumor"[All Fields])) OR (("sexual dysfunctions, psychological"[MeSH Terms] OR ("sexual"[All Fields] AND "dysfunctions"[All Fields] AND "psychological"[All Fields]) OR "psychological sexual dysfunctions"[All Fields] OR ("sexual"[All Fields] AND "dysfunction"[All Fields]) OR "sexual dysfunction"[All Fields] OR "sexual dysfunction, physiological"[MeSH Terms] OR ("sexual"[All Fields] AND "dysfunction"[All Fields] AND "physiological"[All Fields]) OR "physiological sexual dysfunction"[All Fields] OR ("sexual"[All Fields] AND "dysfunction"[All Fields])) AND ("neuroendocrine tumour"[All Fields] OR "neuroendocrine tumors"[MeSH Terms] OR ("neuroendocrine"[All Fields] AND "tumors"[All Fields]) OR "neuroendocrine tumors"[All Fields] OR ("neuroendocrine"[All Fields] AND "tumor"[All Fields]) OR "neuroendocrine tumor"[All Fields])) OR (("erectility"[All Fields] OR "penile erection"[MeSH Terms] OR ("penile"[All Fields] AND "erection"[All Fields]) OR "penile erection"[All Fields] OR "erectile"[All Fields]) AND ("functional"[All Fields] OR "functional s"[All Fields] OR "functionalities"[All Fields] OR "functionality"[All Fields] OR "functionalization"[All Fields] OR "functionalizations"[All Fields] OR "functionalize"[All Fields] OR "functionalized"[All Fields] OR "functionalizes"[All Fields] OR "functionalizing"[All Fields] OR "functionally"[All Fields] OR "functionals"[All Fields] OR "functioned"[All Fields] OR "functioning"[All Fields] OR "functionings"[All Fields] OR "functions"[All Fields] OR "physiology"[MeSH Subheading] OR "physiology"[All Fields] OR "function"[All Fields] OR "physiology"[MeSH Terms]) AND ("neuroendocrin"[All Fields] OR "neuroendocrinal"[All Fields] OR "neuroendocrines"[All Fields] OR "neurosecretory systems"[MeSH Terms] OR ("neurosecretory"[All Fields] AND "systems"[All Fields]) OR "neurosecretory systems"[All Fields] OR "neuroendocrine"[All Fields])) OR (("erectile

dysfunction"[MeSH Terms] OR ("erectile"[All Fields] AND "dysfunction"[All Fields]) OR "erectile dysfunction"[All Fields]) AND ("neuroendocrin"[All Fields] OR "neuroendocrinal"[All Fields] OR "neuroendocrines"[All Fields] OR "neurosecretory systems"[MeSH Terms] OR ("neurosecretory"[All Fields] AND "systems"[All Fields]) OR "neurosecretory systems"[All Fields] OR "neuroendocrine"[All Fields])) OR ("177"[All Fields] AND "lu-dotatate"[All Fields] AND ("quality of life"[MeSH Terms] OR ("quality"[All Fields] AND "life"[All Fields]) OR "quality of life"[All Fields])) OR ("177"[All Fields] AND ("lutetium"[MeSH Terms] OR "lutetium"[All Fields]) AND ("copper dotatate cu 64"[Supplementary Concept] OR "copper dotatate cu 64"[All Fields] OR "dotatate"[All Fields]) AND ("quality of life"[MeSH Terms] OR ("quality"[All Fields] AND "life"[All Fields]) OR "quality of life"[All Fields])) OR ("90"[All Fields] AND ("yttrium"[MeSH Terms] OR "yttrium"[All Fields]) AND ("quality of life"[MeSH Terms] OR ("quality"[All Fields] AND "life"[All Fields]) OR "quality of life"[All Fields])) OR ("90"[All Fields] AND ("yttrium"[MeSH Terms] OR "yttrium"[All Fields]) AND (("sexual behavior"[MeSH Terms] OR ("sexual"[All Fields] AND "behavior"[All Fields]) OR "sexual behavior"[All Fields] OR "sexual"[All Fields] OR "sexually"[All Fields] OR "sexualities"[All Fields] OR "sexuality"[MeSH Terms] OR "sexuality"[All Fields] OR "sexualization"[All Fields] OR "sexualize"[All Fields] OR "sexualized"[All Fields] OR "sexualizing"[All Fields] OR "sexuals"[All Fields]) AND ("functional"[All Fields] OR "functional s"[All Fields] OR "functionalities"[All Fields] OR "functionality"[All Fields] OR "functionalization"[All Fields] OR "functionalizations"[All Fields] OR "functionalize"[All Fields] OR "functionalized"[All Fields] OR "functionalizes"[All Fields] OR "functionalizing"[All Fields] OR "functionally"[All Fields] OR "functionals"[All Fields] OR "functioned"[All Fields] OR "functioning"[All Fields] OR "functionings"[All Fields] OR "functions"[All Fields] OR "physiology"[MeSH Subheading] OR "physiology"[All Fields] OR "function"[All Fields] OR "physiology"[MeSH Terms]))) OR ("90"[All Fields] AND ("yttrium"[MeSH Terms] OR "yttrium"[All Fields]) AND ("sexual dysfunctions, psychological"[MeSH Terms] OR ("sexual"[All Fields] AND "dysfunctions"[All Fields] AND "psychological"[All Fields]) OR "psychological sexual dysfunctions"[All Fields] OR ("sexual"[All Fields] AND "dysfunction"[All Fields]) OR "sexual dysfunction"[All Fields] OR "sexual

dysfunction, physiological"[MeSH Terms] OR ("sexual"[All Fields] AND "dysfunction"[All Fields] AND "physiological"[All Fields]) OR "physiological sexual dysfunction"[All Fields] OR ("sexual"[All Fields] AND "dysfunction"[All Fields]))) OR ("177"[All Fields] AND "lu-dotatate"[All Fields] AND (("erectility"[All Fields] OR "penile erection"[MeSH Terms] OR ("penile"[All Fields] AND "erection"[All Fields]) OR "penile erection"[All Fields] OR "erectile"[All Fields]) AND ("functional"[All Fields] OR "functional s"[All Fields] OR "functionalities"[All Fields] OR "functionality"[All Fields] OR "functionalization"[All Fields] OR "functionalizations"[All Fields] OR "functionalize"[All Fields] OR "functionalized"[All Fields] OR "functionalizes"[All Fields] OR "functionalizing"[All Fields] OR "functionally"[All Fields] OR "functionals"[All Fields] OR "functioned"[All Fields] OR "functioning"[All Fields] OR "functionings"[All Fields] OR "functions"[All Fields] OR "physiology"[MeSH Subheading] OR "physiology"[All Fields] OR "function"[All Fields] OR "physiology"[MeSH Terms]))) OR ("177"[All Fields] AND ("lutetium"[MeSH Terms] OR "lutetium"[All Fields]) AND ("copper dotatate cu 64"[Supplementary Concept] OR "copper dotatate cu 64"[All Fields] OR "dotatate"[All Fields]) AND (("erectility"[All Fields] OR "penile erection"[MeSH Terms] OR ("penile"[All Fields] AND "erection"[All Fields]) OR "penile erection"[All Fields] OR "erectile"[All Fields]) AND ("functional"[All Fields] OR "functional s"[All Fields] OR "functionalities"[All Fields] OR "functionality"[All Fields] OR "functionalization"[All Fields] OR "functionalizations"[All Fields] OR "functionalize"[All Fields] OR "functionalized"[All Fields] OR "functionalizes"[All Fields] OR "functionalizing"[All Fields] OR "functionally"[All Fields] OR "functionals"[All Fields] OR "functioned"[All Fields] OR "functioning"[All Fields] OR "functionings"[All Fields] OR "functions"[All Fields] OR "physiology"[MeSH Subheading] OR "physiology"[All Fields] OR "function"[All Fields] OR "physiology"[MeSH Terms]))) OR ("177"[All Fields] AND "lu-dotatate"[All Fields] AND ("erectile dysfunction"[MeSH Terms] OR ("erectile"[All Fields] AND "dysfunction"[All Fields]) OR "erectile dysfunction"[All Fields])) OR ("177"[All Fields] AND ("lutetium"[MeSH Terms] OR "lutetium"[All Fields]) AND ("copper dotatate cu 64"[Supplementary Concept] OR "copper dotatate cu 64"[All Fields] OR "dotatate"[All Fields]) AND ("erectile dysfunction"[MeSH Terms] OR ("erectile"[All Fields] AND "dysfunction"[All Fields]) OR "erectile dysfunction"[All Fields])) OR ("177"[All

Fields] AND ("lutetium"[MeSH Terms] OR "lutetium"[All Fields]) AND ("copper dotatate cu 64"[Supplementary Concept] OR "copper dotatate cu 64"[All Fields] OR "dotatate"[All Fields]) AND ("sexual dysfunctions, psychological"[MeSH Terms] OR ("sexual"[All Fields] AND "dysfunctions"[All Fields] AND "psychological"[All Fields]) OR "psychological sexual dysfunctions"[All Fields] OR ("sexual"[All Fields] AND "dysfunction"[All Fields]) OR "sexual dysfunction"[All Fields] OR "sexual dysfunction, physiological"[MeSH Terms] OR ("sexual"[All Fields] AND "dysfunction"[All Fields] AND "physiological"[All Fields]) OR "physiological sexual dysfunction"[All Fields] OR ("sexual"[All Fields] AND "dysfunction"[All Fields]))) OR ("177"[All Fields] AND "lu-dotatate"[All Fields] AND (("sexual behavior"[MeSH Terms] OR ("sexual"[All Fields] AND "behavior"[All Fields]) OR "sexual behavior"[All Fields] OR "sexual"[All Fields] OR "sexually"[All Fields] OR "sexualities"[All Fields] OR "sexuality"[MeSH Terms] OR "sexuality"[All Fields] OR "sexualization"[All Fields] OR "sexualize"[All Fields] OR "sexualized"[All Fields] OR "sexualizing"[All Fields] OR "sexuals"[All Fields]) AND ("functional"[All Fields] OR "functional s"[All Fields] OR "functionalities"[All Fields] OR "functionality"[All Fields] OR "functionalization"[All Fields] OR "functionalizations"[All Fields] OR "functionalize"[All Fields] OR "functionalized"[All Fields] OR "functionalizes"[All Fields] OR "functionalizing"[All Fields] OR "functionally"[All Fields] OR "functionals"[All Fields] OR "functioned"[All Fields] OR "functioning"[All Fields] OR "functionings"[All Fields] OR "functions"[All Fields] OR "physiology"[MeSH Subheading] OR "physiology"[All Fields] OR "function"[All Fields] OR "physiology"[MeSH Terms]))) OR ("90"[All Fields] AND ("yttrium"[MeSH Terms] OR "yttrium"[All Fields]) AND ("erectile dysfunction"[MeSH Terms] OR ("erectile"[All Fields] AND "dysfunction"[All Fields]) OR "erectile dysfunction"[All Fields])) OR ("90"[All Fields] AND ("yttrium"[MeSH Terms] OR "yttrium"[All Fields]) AND (("erectility"[All Fields] OR "penile erection"[MeSH Terms] OR ("penile"[All Fields] AND "erection"[All Fields]) OR "penile erection"[All Fields] OR "erectile"[All Fields]) AND ("functional"[All Fields] OR "functional s"[All Fields] OR "functionalities"[All Fields] OR "functionality"[All Fields] OR "functionalization"[All Fields] OR "functionalizations"[All Fields] OR "functionalize"[All Fields] OR "functionalized"[All Fields] OR "functionalizes"[All Fields] OR "functionalizing"[All Fields] OR "functionally"[All

Fields] OR "functionals"[All Fields] OR "functioned"[All Fields] OR "functioning"[All Fields] OR "functionings"[All Fields] OR "functions"[All Fields] OR "physiology"[MeSH Subheading] OR "physiology"[All Fields] OR "function"[All Fields] OR "physiology"[MeSH Terms]))) OR ("90"[All Fields] AND ("yttrium"[MeSH Terms] OR "yttrium"[All Fields]) AND ("sexual dysfunctions, psychological"[MeSH Terms] OR ("sexual"[All Fields] AND "dysfunctions"[All Fields] AND "psychological"[All Fields]) OR "psychological sexual dysfunctions"[All Fields] OR ("sexual"[All Fields] AND "dysfunction"[All Fields]) OR "sexual dysfunction"[All Fields] OR "sexual dysfunction, physiological"[MeSH Terms] OR ("sexual"[All Fields] AND "dysfunction"[All Fields] AND "physiological"[All Fields]) OR "physiological sexual dysfunction"[All Fields] OR ("sexual"[All Fields] AND "dysfunction"[All Fields]))) OR ("90"[All Fields] AND ("yttrium"[MeSH Terms] OR "yttrium"[All Fields]) AND (("sexual behavior"[MeSH Terms] OR ("sexual"[All Fields] AND "behavior"[All Fields]) OR "sexual behavior"[All Fields] OR "sexual"[All Fields] OR "sexually"[All Fields] OR "sexualities"[All Fields] OR "sexuality"[MeSH Terms] OR "sexuality"[All Fields] OR "sexualization"[All Fields] OR "sexualize"[All Fields] OR "sexualized"[All Fields] OR "sexualizing"[All Fields] OR "sexuals"[All Fields]) AND ("functional"[All Fields] OR "functional s"[All Fields] OR "functionalities"[All Fields] OR "functionality"[All Fields] OR "functionalization"[All Fields] OR "functionalizations"[All Fields] OR "functionalize"[All Fields] OR "functionalized"[All Fields] OR "functionalizes"[All Fields] OR "functionalizing"[All Fields] OR "functionally"[All Fields] OR "functionals"[All Fields] OR "functioned"[All Fields] OR "functioning"[All Fields] OR "functionings"[All Fields] OR "functions"[All Fields] OR "physiology"[MeSH Subheading] OR "physiology"[All Fields] OR "function"[All Fields] OR "physiology"[MeSH Terms]))))

2. EORTC QLQ-C30 and GINET21 questionnaires

The EORTC QLQ-C30 consists of 30 items: The first 28 questions have four possible answers (1 = not at all; 2 = a little; 3 = quite a bit; 4 = very much), while the response format of the last two questions is a 7-point Likert scale. Questions 1–5 are dedicated to the perception of physical forces in relation to routine activities such as taking a short walk, eating or dressing, or activities that require

greater physical effort, such as lifting heavy objects (shopping bag or suitcase) or going on a long walk. Questions 6 and 7 are dedicated to the limitations in working or in performing daily activities and hobbies. Items 8–12 are related to general problems like presence of pain, shortness of breath, insomnia, or asthenia. Questions 13–18 focus on the gastrointestinal symptoms present in many cancers, especially because of the treatments administered. Questions 19–25 investigate the psychological aspect of the patient, focusing on the interference between pain and daily activities and perceived psychic well-being: tension, anxiety, irritability, depression, or memory re-duction. Questions 26–28 investigate the perceived impact of the disease on family, social, and financial life. Finally, the last two questions, rated on a 7-point Likert scale, aim at assessing the patient's state of health and quality of life as a whole [12].

The QLQ-GINET21 contains a total of 21 items whose answers are expressed as a 4-point Likert scale (1 = not at all; 2 = a little; 3 = quite a bit; 4 = very much or not applicable). It consists of three defined multi-item symptom scales (endocrine – three questions; gastrointestinal – five questions; and treatment-related side effects – three questions), two single-item symptoms (bone/muscle pain and concern about weight loss or gain), two psychosocial scales (social function – three questions; disease-related worries – three questions), and two other single items (sexuality and communication) [13]. Both questionnaires can be performed quickly, about 10–15 minutes on average each, and most patients do not require assistance with completion because the questions are very clear, with a low rate of missing values.
